# Parasite escape mechanisms drive morphological diversification in avian lice

**DOI:** 10.1098/rspb.2023.2665

**Published:** 2024-03-27

**Authors:** Stanislav Kolencik, Edward L. Stanley, Aswaj Punnath, Avery R. Grant, Jorge Doña, Kevin P. Johnson, Julie M. Allen

**Affiliations:** ^1^ Department of Biology, University of Nevada Reno, Reno, NV 89557, USA; ^2^ Faculty of Mathematics, Natural Sciences, and Information Technologies, University of Primorska, Glagoljaška 8, 6000 Koper, Slovenia; ^3^ Department of Natural History, Florida Museum of Natural History, University of Florida, Gainesville, FL 32611, USA; ^4^ Entomology and Nematology Department, University of Florida, Gainesville, FL 32611, USA; ^5^ Illinois Natural History Survey, Prairie Research Institute, University of Illinois at Urbana Champaign, Champaign, IL 61820, USA; ^6^ Departamento de Biología Animal, Universidad de Granada, 18071 Granada, Spain; ^7^ Department of Biological Sciences, Virginia Tech, Blacksburg, VA 24061, USA

**Keywords:** bird lice, co-adaptations, micro-morphology, CT scanning, evolution

## Abstract

Organisms that have repeatedly evolved similar morphologies owing to the same selective pressures provide excellent cases in which to examine specific morphological changes and their relevance to the ecology and evolution of taxa. Hosts of permanent parasites act as an independent evolutionary experiment, as parasites on these hosts are thought to be undergoing similar selective pressures. Parasitic feather lice have repeatedly diversified into convergent ecomorphs in different microhabitats on their avian hosts. We quantified specific morphological characters to determine (i) which traits are associated with each ecomorph, (ii) the quantitative differences between these ecomorphs, and (iii) if there is evidence of displacement among co-occurring lice as might be expected under louse–louse competition on the host. We used nano-computed tomography scan data of 89 specimens, belonging to four repeatedly evolved ecomorphs, to examine their mandibular muscle volume, limb length and three-dimensional head shape data. Here, we find evidence that lice repeatedly evolve similar morphologies as a mechanism to escape host defences, but also diverge into different ecomorphs related to the way they escape these defences. Lice that co-occur with other genera on a host exhibit greater morphological divergence, indicating a potential role of competition in evolutionary divergence.

## 1. Introduction

Animals are hosts to many different groups of parasites. From the parasite's perspective, hosts represent isolated environments that contain finite resources distributed across a variety of ecological niches. As such, they closely mirror the classic ‘island’ systems that have formed the basis of many foundational studies of the field of evolutionary biology (e.g. greater Antillean *Anolis* lizards [1], African rift lake cichlids [2] and Hawaiian honeycreepers [3]). While many islands are relatively young in comparison with the continents, some have been isolated for long periods. The isolation and high habitat heterogeneity in these island systems often result in ecologically specialized flora and fauna that have diversified and radiated *in situ* following colonization [4,5]. In these cases, competition over finite resources leads to niche partitioning—a division of resources in an ecosystem by different species to avoid competition, creating divergent selective pressures that propel a single phenotype into a series of specialized ecomorphs within an island [4]. This process can repeat across isolated systems that contain similar niches, resulting in similar evolutionary shifts (convergence) of ecologically specialized phenotypes. Permanent parasites are subject to many of the same selective pressures as these classic island model systems [6,7], but face the additional challenge of a dynamically evolving host defence system [8]. Therefore, these systems may provide novel insights into understanding how ecological pressures drive macroevolutionary patterns. However, applying a quantitative approach to studying ecomorphological diversification and convergence in parasites has often been impeded owing to their relatively small size and difficulties in direct observation.

Avian feather lice are a group of permanent ectoparasites that show evidence of repeated convergence in ecomorphological features in response to host defence [9]. Because lice spend their entire life cycle on a single host and do not fly or jump, they often have limited opportunities to disperse to other species of birds, similar to the island systems, which might promote *in situ* diversification [8,10]. Some birds are host to multiple species of lice [11], and competition between lice could result in resource partitioning and subsequent microhabitat specialization. Feather lice feed mostly on feathers [11]. Thus, a high louse infestation rate can cause significant feather damage resulting in the lowered ability of the host to thermoregulate and may result in secondary infections and symptoms such as restlessness, anorexia, weight loss, reduced egg laying and even death of the infected bird [11,12]. Because of these factors, birds and lice are trapped in a coevolutionary system where birds have evolved defences to remove ectoparasites, and conversely, lice have coevolved counter-adaptations to host defences to remain attached to the host.

**Figure 1.**
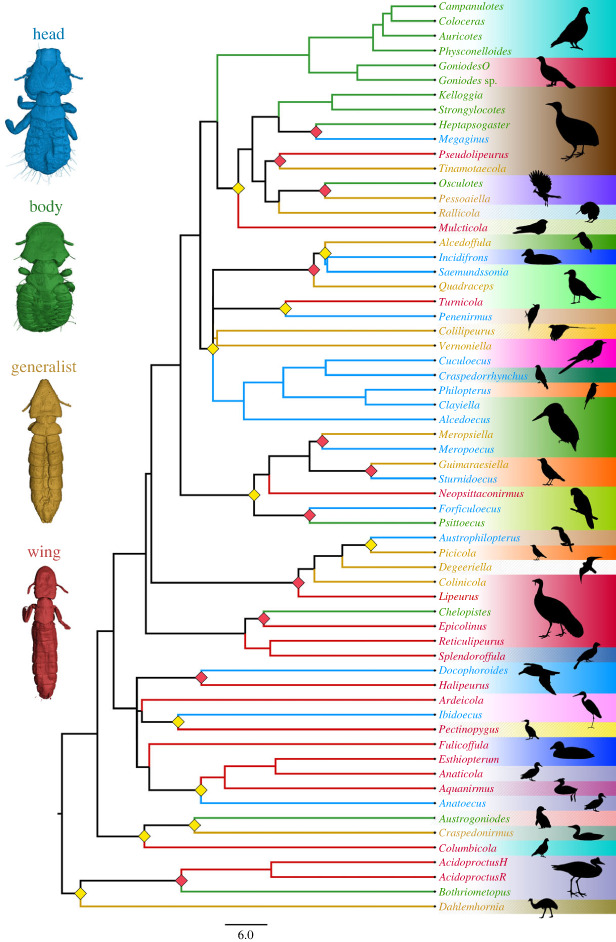
Phylogenomic relationships between feather louse genera belonging to four ecomorph groups. Specimens and branches are coloured according to their ecomorph identification. The background colours represent the different bird host orders. The diamond shape on the nodes represents ecomorph transitions between sister taxa (red for lice parasitizing from the same host order, and yellow for lice parasitizing different host orders). *AcidoproctusH,* *Acidoproctus hilli*; *AcidoproctusR*, *Acidoproctus rostratus*; *GoniodesO*, *Goniodes ortygis*.

Birds have two main defences against lice: preening with the bill and scratching with the feet [13]. Feather lice (Phthiraptera: Ischnocera) have evolved into different ecomorphs (figure 1) depending on the part of the host's body they occupy [9,14–16] and how they escape from host defences. The most common ecomorphs found across feather lice are termed wing, head, body and generalist lice [9,14]. Wing lice are long and slender in shape and insert between the barbs of the bird's wing feathers, making it difficult for the bird to remove them through preening [17]. Head lice have a plump, rounded body with a triangular head, likely supporting strong mandibular muscularization (the chewing muscles). These lice remain on the head where the bird cannot preen with its bill, and it is thought they use this muscularization to bite down and hold onto feather barbs to avoid being removed by scratching [14,18]. Body lice have a short, rounded body and head, and escape host defences by burrowing into the downy parts of the feathers on the body of the bird [19]. Some feather lice are more generalized in their body form and can be found over many regions of the host [14]. Categorization of the microhabitat specialization of lice has been based on observational evidence [14,15] and qualitative assessments of the general morphological form [16]. A detailed study characterizing and quantifying the morphological features specific to each ecomorph and analysing their differences has not been done. Quantitative assessments of functional traits have advantages over a purely categorical approach [20] as they allow a more nuanced assessment of morphological disparity and enable hypothesis testing to understand the processes behind the different morphological features of each ecomorph.

Traditional morphological analyses of lice have used slide-mounted specimens and microscopy to find morphological differences between species and to make detailed descriptions of new species (e.g. [21]). This method can identify the presence or absence of some characteristic structures and recover two-dimensional (2D) measurements, such as length and width, but is limited at capturing linear measurements in a complex three-dimensional (3D) environment (e.g. depth) and cannot recover volumetric data. Nano-computed tomography (nano-CT) uses X-ray imaging to reconstruct a high-resolution, 3D density map of an object. This allows the isolation and visualization of external and internal features and facilitates the quantification and categorization of complex 3D structures. Recent advances in the availability of 3D imaging have enabled quantitative measurements and analyses of even small parasites [22,23].

Here, we collected and analysed nano-CT scan data from different louse species representing several independent evolutionary origins of each ecomorph (head, body, wing and generalist). First, because ecomorphs have traditionally been assigned with qualitative assessments [16], we wanted to determine if each ecomorph resides in its own morphospace and can be assigned using quantitative landmark data. Next, we investigated the hypothesis that convergence in the overall morphology of feather louse ecomorphs has resulted from the same specific morphological transitions [9]. We focused on two features that are believed to be adaptive for each ecomorph in escaping host defence. First, it has been hypothesized that the main counter-defence of head lice to avoid being removed by scratching is to slide a feather barb into the cranial rostral groove and bite with the mandibles to remain attached [9,14]. Therefore, large mandibular muscles might be favoured and could be the reason for convergently evolved head lice to have an expanded temple region compared with other ecomorphs. Second, we predicted differences in proportional lengths of the leg parts (tibia and femur), between more mobile (e.g. generalists) and sedentary ecomorphs (e.g. head) [14]. Additionally, wing lice, in some cases, use phoresis (attaching to another organism to travel) on hippoboscid flies as a way of moving between the hosts [24,25]; thus, it is likely that their leg morphology may affect their ability to grasp onto the flies or birds.

Lastly, previous studies have suggested that lice partition different microhabitats on their hosts owing to interspecific competition [26]. If competition is a significant driver of habitat partitioning and subsequent ecological adaptations, we expect lice that co-occur with other louse species on the same host to have more extreme phenotypes and higher rates of evolution than those that occupy the host alone.

## 2. Results

Specimens of 89 feather lice representing 62 species were included in our analyses. Our samples consisted of 21 body, 20 generalist, 22 head and 26 wing louse specimens, representing 22 ecomorph transitions within the Ischnocera (figure 1). We analysed four different datasets: (1) two geometric morphometric analyses using head shape landmark data, one with a newly reconstructed phylogenomic tree and one without; (2) the proportional volume of the chewing muscles; (3) the proportional lengths of legs; and (4) the rate of evolution between lice that cohabit with other lice on the same host species and those that are the sole louse parasites on their host species.

### (a) Quantitative ecomorph identification

#### (i) Head shape

**Figure 2.**
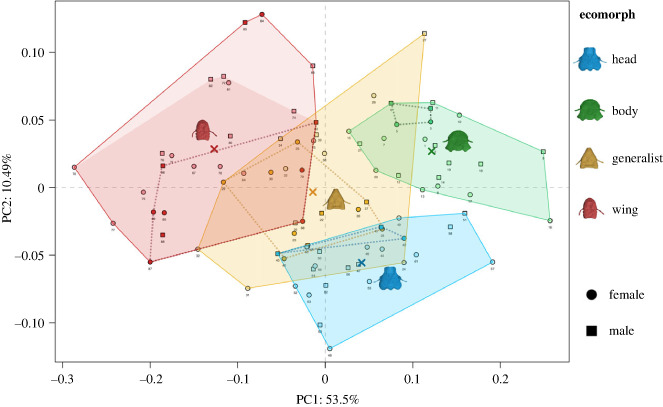
Feather louse morphospace. Principal component analysis (PCA) results of generalized Procrustes analysis of 14 landmark data representing the head shape of 88 specimens belonging to four ecomorph groups (body, generalist, head and wing). PC1 (*x*) explains 53.5% of variability; PC2 (*y*) explains 10.49%. The coloured polygons represent the ecomorph group morphospace, with the mean PC1 and PC2 values for each ecomorph marked by an ×. The brighter red background colour polygon shows data including three *Acidoproctus* specimens (points 64–66), with the unusual head shape making their representation in the wing ecomorph group unclear. The dotted lines represent the space in which specimens (darker points) that do not co-occur with other ecomorphs on their hosts are found. The points outside those dotted lines represent lice that co-occur with other lice species on the same host, demonstrating that they reside in a more extreme morphospace, whereas all the lice that do not co-occur are found closer to the means of each ecomorph space.

**Figure 3.**
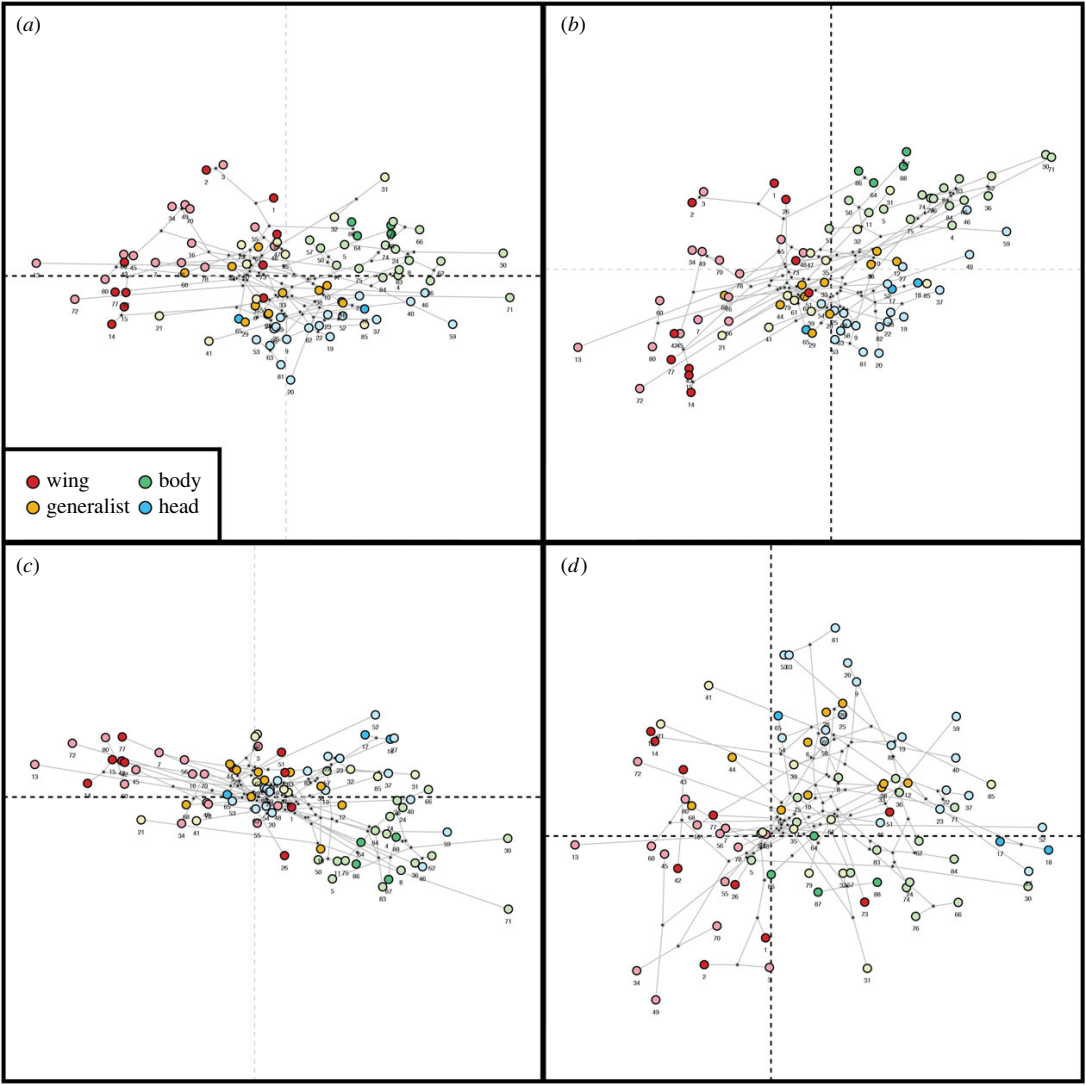
Phylo-morphospace analysis of louse ecomorphs. (*a*) A phylogenetic principal component analysis (phy-PCA) with centring and projections via ordinary least squares (OLS); (*c*) phy-PCA with centring and projections via generalized-least squares (GLS); (*b*) a phylogenetically aligned PCA (PACA) with OLS; (*d*) PACA with GLS.

Three habitat specialist ecomorphs (wing, body and head) were separated into non-overlapping morphospace, while generalist lice appeared in an intermediate region of morphospace between and overlapping each of the other three groups (figure 2). Ecomorph type was significantly associated with the head shape when accounting for the phylogenetic non-independence of the data (phylogenetic ANOVA of PC1 and PC2). The significant group-wise differences found in PC1 (*F* = 53.68, *p* < 0.001) were between wing and head, wing and body (*p* < 0.001), wing and generalist, and body and generalist (*p* < 0.01). Comparing PC2 (*F* = 24.1, *p* < 0.001), significant group-wise differences were found between the head and all other ecomorph groups (*p* < 0.01). Interestingly, while the head shape exhibited a significant phylogenetic signal, this was less than expected under a Brownian motion model of evolution (*K*_mult_ = 0.697, *p* < 0.001). Indeed, this result is congruent with the topology of the phylogenetic tree (figure 1), in which there were multiple cases of sister taxa belonging to different ecomorph groups. Similarly, the results of our phylo-morphospace analysis, which combined the principal component analysis (PCA) with phylogenetic tree data, showed that sister taxa were often associated with different morphospaces (figure 2). Here, the phylogenetically aligned PCA (PACA), especially when using generalized-least squares (GLS)-centring and estimation, appears to affect the ecomorph grouping the most (figure 3*d*).

Several species that were identified as habitat specialists were recovered in areas of morphospace occupied by generalist ecomorphs (e.g. the wing louse *Aquanirmus occidentalis*; point 68 in figure 2), while some ‘generalist‘ lice were found in wing louse (*Dahlemhornia asymmetrica*; point 29 in figure 2) or head louse (*Guimaraesiella antiqua*; point 24 in figure 2) morphospace. Interestingly, a species from the *Brueelia*-complex, *G. antiqua*, has an unusually large mandibular muscularization (electronic supplementary material, dataset S1). Additionally, the centroid of the head ecomorphs was located within the overlapping space of the generalist morphospace, but all other ecomorph groups had their mean values in their own discrete morphospace (marked with ‘×’ in figure 2). The remaining generalists that overlapped with the head ecomorph were found along the border of these two groups, and adding additional morphological characters might help to separate them further. The specimen *Megaginus tataupensis* (point 57 in figure 2), which was originally assigned as a body louse (with other members of tinamou body lice), fell into the head louse morphospace. Similarly, *Bothriometopus macrocnemis* (points 5 and 6 in figure 2), originally characterized as a wing louse, appeared within the body louse morphospace. These lice specifically had a combination of factors that suggest the original ecomorph assignment is possibly not accurate, whereas others in overlapping morphospace require further analysis.

### (b) Ecomorph-specific character differences

#### (i) Chewing muscles

**Figure 4.**
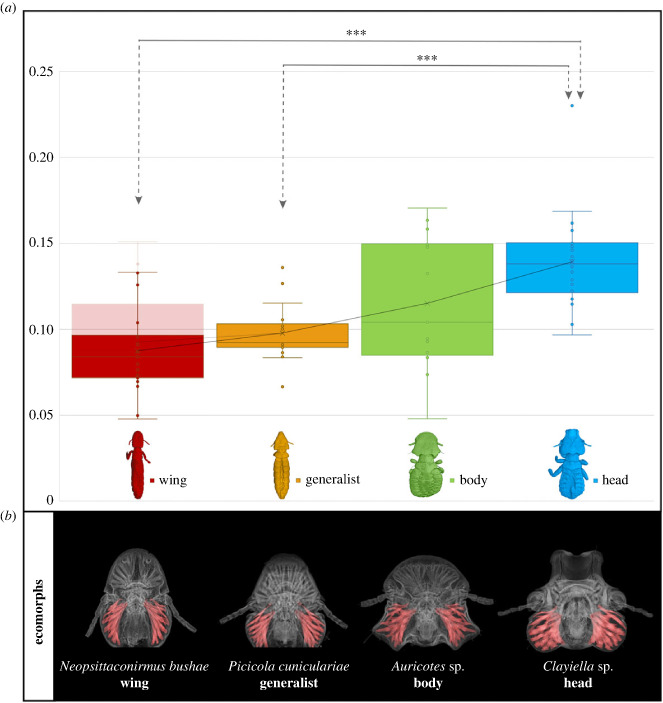
Mandibular muscularization of feather louse ecomorphs. (*a*) Proportional volume of the chewing muscles to the volume of the head (*N* = 77, or 79 with *Acidoproctus*). Asterisks indicate significant differences between group comparisons (***p < 0.001). The pink colour shows data including two *Acidoproctus* specimens, which have a strongly indented marginal carina between frontal projections, affecting the total volume of the head. (*b*) CT scan image of the head of four ecomorph groups with the mandibular muscles highlighted in pink.

We found differences in the relative proportion of chewing muscles between ecomorphs, with head lice having the largest and wing lice the smallest proportional mandibular muscularization (figure 4; electronic supplementary material, figure S1). Chewing muscle proportional volume was significantly associated with ecomorph (Kruskal–Wallis (KW) test, *H* = 25.324, d.f. = 3, *p* < 0.001; electronic supplementary material S2*a*), whereas the sex of the specimen did not have a significant impact (*H* = 0.283, d.f. = 1, *p* > 0.05; electronic supplementary material, figure S1). Significant group-wise differences were found between head and wing ecomorphs, and between head and generalist ecomorphs (figure 4; electronic supplementary material, figure S2*b*). When accounting for the phylogenetic non-independence of the data, we found that ecomorph still accounts for a significant amount of variation in the volume of the mandibular muscularization (*F* = 12.84, *p* < 0.001), with the same significant groupings between head and wing, and head and generalist lice.

Two species from the wing louse genus *Acidoproctus* had the highest proportional volume of chewing muscles compared with other wing lice. *Acidoproctus* has an unusual head shape, with a strongly indented marginal carina between frontal projections, which reduces the total volume of the head and thus results in a higher proportional volume of chewing muscles. Thus, we also removed *Acidoproctus* species for comparison as it seems to be functioning differently from other wing lice (figure 4; electronic supplementary material, figure S1).

#### (ii) Leg length

**Figure 5.**
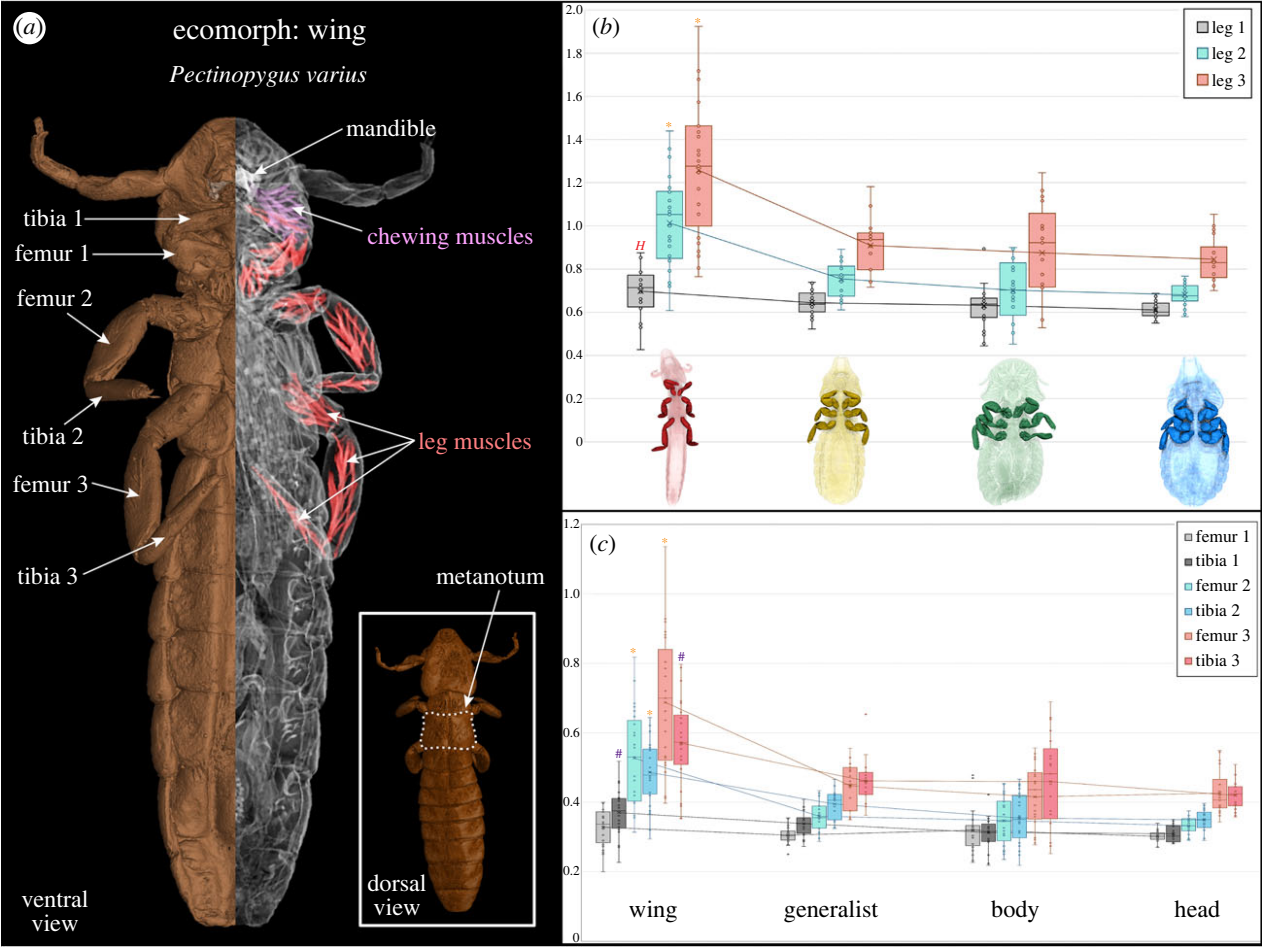
Proportional length of legs for each ecomorph group. (*a*) Illustrated louse morphology; (*b*) proportional length of legs (femur and tibia together) to metanotum width; (*c*) proportional length of leg parts (femur and tibia separately) to metanotum width. Asterisk shows significant differences between wing ecomorph and all other groups; hash shows significant differences between the wing and both head and body groups; the letter *H* shows a significant difference between wing and head ecomorphs.

We found a significant difference in the proportional length of the legs between ecomorph groups, both when including all six leg segments and for the whole leg combined (KW test, *p* < 0.001; figure 5; electronic supplementary material, figures S3 and S4). When including all six leg segments in a Dunn's *post hoc* test, significant group-wise differences in proportional length were found between wing and generalist lice, and between both wing and body, and wing and head lice. The femur of the first pair of legs was not significantly different between ecomorphs. However, when focusing on the whole legs (femur + tibia), all three legs were significantly different between ecomorphs (electronic supplementary material, figures S3 and S4).

### (c) Solo versus co-occurring lice

We scored each species of the louse as ‘solo’ if there were no records of other species of lice found on their host species, or ‘co-occurring’ if there were records of other feather louse species on their same hosts. Across all groups, co-occurring lice had significantly higher evolutionary rates than solo species (*p* = 0.0053, evolutionary rate of co-occurring species = 3.413 × 10^−6^, rate for solo species = 2.544 × 10^−6^, observed rate ratio = 1.342, effect size = 2.41) and this was not significantly different between ecomorph groups (*p* > 0.05, observed rate ratio = 1.657, effect size = 0.382). Further, co-occurring lice were found in more extreme morphospace than their solo counterparts (figure 2).

## 3. Discussion

Repeated convergent evolution in systems with similar selective pressures can help to illuminate the link between evolutionary patterns, ecological processes and adaptations. As has been extensively categorized in island radiations, ectoparasites may partition host habitat space and repeatedly evolve into ecomorphs as they adapt to that space. For parasites, this adaptation often involves responding to host defences. Here, we examined specific morphological differences between convergently evolved ecomorphs of avian feather lice. We found evidence for intra-host competition leading to niche partitioning and rapid evolution of more extreme phenotypes that differ in how they respond to host defences.

### (a) Host defences and louse adaptations

Experimental transfer experiments have demonstrated the ability of lice to rapidly adapt to host habitats [27], supporting our results. Studies have also shown the rapid evolution of the colorations of lice in response to host defence. Bush *et al*. [16] investigated the ability of feather lice to blend in with their surroundings (crypsis) and found that lice living on areas of the bird's body that the host can see (e.g. wing) have evolved background matching coloration to avoid being seen by the host. Other experimental transfer experiments have demonstrated the rapid evolution of coloration by placing lice on light and dark hosts for a short period of time [28]. In view of these experiments, it is reasonable to suspect that morphological adaptation to escape from host defences could evolve relatively rapidly, depending on the part of the host's body where this escape occurs. For example, wing lice transferred from large pigeons to smaller doves move onto the head to escape from host preening because they are too large to fit between the smaller interbarb spaces of smaller doves [29].

Geometric morphometric analysis of head shape reveals that head, body and wing ecomorphs occupy distinct regions of morphospace, with generalist lice intermediate between these groups (figure 2). These results agree to some degree with a hypothesis that differences in the relative volume of chewing muscles will result in different ecomorph head shapes, with the posterior part of the head (where the mandibular muscularization is located) becoming expanded in species with large chewing muscles. Head lice are thought to bite down to grip onto feather barbs to avoid being removed by scratching and, therefore, require larger chewing muscles than other ecomorphs [9,14]. As predicted, we found that head lice have significantly larger proportional mandibular muscularization, followed by body, generalist and wing lice (figure 4). The diet of all feather lice is very similar [11] in that all feather lice eat the downy portions of feathers, so it is reasonable to suggest that the major differences in chewing muscles are related to escaping host defences. In contrast to head lice, wing lice hide between the barbs of the wing feathers to avoid being removed by preening and do not grip these barbs with their mandibles, given the large size of the feather barbs in relation to louse size. To insert into this interbarb space, wing lice must be long and slender, and thus the size of their mandibular muscularization is likely constrained because they would need expanded temples to accommodate larger muscles, as seen in head louse ecomorphs (e.g. figure 4).

In the analysis of the proportional mandibular muscularization, the most significant differences were found between head and wing and between head and generalist ecomorphs. Interestingly, no significant differences were found between body lice and other groups. Body lice had the largest variance in the proportional volume of the chewing muscles (figure 4) and seem to be more variable in morphology than initially expected. However, when accounting for the phylogenetic non-independence of the head shape landmark data, head lice differ significantly in PC2 values from all other ecomorphs, including body lice (*p* < 0.05). Thus, even while there was a high variance in chewing muscle volume in body louse ecomorphs, they still seem to occupy a morphospace distinct from head lice. Moreover, these results suggest that there are additional morphological differences in head features between the ecomorph groups other than chewing muscles. This group likely needs additional attention to understand their full range of morphological variation and whether this relates to variation in escape strategy or to some other ecological difference. Lastly, most generalists occupy an area of morphospace intermediate to the more specialized ecomorphs, perhaps mirroring their ability to traverse the body and wing on the host.

A few generalist species have unusual head shapes for the group to which they have been assigned. *Colinicola mearnsi* occupies a unique area of head shape morphospace, closer to the body louse morphospace. *Guimaraesiella antiqua* is relatively close to the mean shape of the head louse (marked as a blue ‘×’ in figure 2) and has a strong mandibular muscularization. Interestingly, some species from the *Brueelia*-complex are known for their phoretic capabilities on hippoboscid flies; however, they bite down on the fly to hold on rather than using legs like the wing lice [30]. This aspect could explain their need for stronger mandibular muscularization for successful host switching. While the emu louse *Dahlemhornia* appears to be well within the wing morphospace, this genus has an asymmetrical head, potentially affecting the head shape analysis. These results suggest there are additional selective pressures on these shapes that warrant further study.

As previously mentioned, body lice have a high variability of chewing muscle volumes, and, similarly, there appear to be at least two different groupings of head shape in this group. One of these comprises ‘typical’ body lice with a well-rounded head, e.g. the genus *Goniodes*. Another group of ‘atypical’ body lice includes the genera *Bothriometopus*, *Strongylocotes* and *Kelloggia*, which have head shapes more reminiscent of those from the generalist group. However, these genera have a strong mandibular muscularization (coefficient value 0.13–0.17), well above most generalist lice (0.08–0.11; electronic supplementary material, dataset S1). Interestingly, the closest relative of *Bothriometopus* is the morphologically atypical wing louse genus *Acidoproctus* (figure 1); both can be found on waterfowl, order Anseriformes [31,32]. *Strongylocotes* and *Kelloggia* are tinamou lice members of a single clade (Heptapsogasteridae) [33]. This morphologically diverse group was generally assumed to be composed of body lice but might contain more variability than previously expected. For example, another tinamou louse in Heptapsogasteridae, *Megaginus tataupensis*, was initially assigned to the body louse category [34]. However, our analysis suggests that this species is more appropriately assigned to the head louse group. The PCA morphospace analysis placed *Megaginus* well within the head louse space (figure 2), and it also has strong mandibular muscularization (figure 4; electronic supplementary material, dataset S1). This genus also has a triangular-shaped head and a rounded body more typical of head lice. Additionally, in the case of the genus *Acidoproctus*, an unusual head shape might be the cause of a higher proportional volume of chewing muscles. While this shows some limitations of our method, these cases are relatively rare, and, in general, using head volume to get a proportional volume of the chewing muscles appears to account for much of the variation in ecomorphology.

### (b) Leg length variability

Limb length is expected to differ between ecomorphs owing to their requirements for locomotion. Head lice are relatively sedentary, adapted to movement on the shorter, narrower feathers of the head and neck, whereas generalist and body lice move around more freely on the host [14]. Wing lice typically need to move from the wing to the body of the bird [8,35]. Focusing on the proportional leg length (femur and tibia separately or combined), the most variable group was the wing ecomorph (figure 5). Wing lice differed significantly from all other ecomorphs when we examined femur or tibia either separately (electronic supplementary material, figure S3) or combined (electronic supplementary material, figure S4). By contrast, body, head and generalist lice feed on the down of the feathers in which they are located and escape from host preening in the same regions where they feed. This need for movement between the wing and body of the bird may explain why wing lice have legs with different proportions from other ecomorphs. In addition, wing lice are known to engage in phoresis on hippoboscid flies to disperse between hosts [24,25]. Harbison & Clayton [36] described how wing lice (*Columbicola columbae*) use their third pair of legs to grasp the fly's leg and posited that their long limbs allow a wider stance, making phoretic dispersal possible. The high variance of leg length in our study confirms the likely importance of a larger proportional length of the second and third pair of legs in some species of wing lice (figure 5). For example, *C. columbae* has a second and third pair of legs with a proportional value well above the average of the group (figure 5; electronic supplementary material, dataset S1). Body lice have not been found to use hippoboscid flies for phoresis [25]. Some genera of head and generalist lice use phoresis (e.g. *Sturnidoecus* and *Brueelia*), but typically they attach to the fly with their mandibles rather than their legs [8]. Finally, considering the separate leg segments, the femur of the first leg did not significantly differ between ecomorphs. By contrast, the tibia differed significantly between wing and body, and between wing and head ecomorphs. This implies that there might be a specific functional adaptation of the tibia in the first pair of legs which further studies may reveal.

While Harbison *et al*. [24] stated that both wing and body lice could initiate phoresis on flies, they found that wing lice had significantly lower detachment rates compared with body lice when flies were walking, grooming or flying. In addition, body lice were found not to be attracted to hippoboscid flies, suggesting this is not a primary mode of transportation [24]. Thus, the dominant variability in femur and tibia length between wing lice and all other ecomorph groups suggests their importance in prolonged attachment during the flight of hippoboscid flies. It is well documented that the long and slender shape of wing lice is an important strategy to avoid host removal by preening, in which lice insert between the barbs of the wing feathers [17,24,35]. Our results suggest the potential importance of the length of the second and third pairs of legs in dispersal.

### (c) Phylogenetic structure of ecomorphs

By taking into account the phylogenetic relationships between feather louse taxa, we were able to test whether the shape of the head is more similar among closely related versus more distantly related lice from the same ecomorph. The phylogenetic ANOVA suggested that the type of ecomorph has a more significant effect on the shape of the head than evolutionary history. Additionally, while the head shape in ischnoceran feather lice does exhibit a significant phylogenetic signal (*p* < 0.001), closely related species were less similar to one another than what was expected under a Brownian motion model of evolution (*K*_mult_ < 1). This pattern is also seen in the phylogenetic tree (figure 1), and in the phylo-morphospace analysis, which combines the PCA with the phylogenetic data (figure 3). In our dataset, we reported 22 ecomorph transitions between the sister taxa, from which half of the cases were lice occurring on the same avian order (figure 1). Moreover, in all cases (figure 3*a–d*), these sister taxa are recovered in distinctly different areas of morphospace. When using PACA with GLS-centring and covariance estimation for phylogenetically aligned components analysis of shape data (figure 3*d*), ecomorphs appear to be more scattered across the ordination plot, reducing the original morphospace partitioning between ecomorphs. Therefore, once evolutionary history is taken into account, the ecomorphs do not group consistently. Considering that there is no significant difference in net rates of evolution between the different ecomorph groups in head shape, this pattern indicates a process of repeated adaptive divergence of these parasites within host groups, and convergence when comparing parasites across host groups.

### (d) Interspecific competition

Our results suggest that interspecific competition may be an influential factor in micro-habitat partitioning among louse species. The head shape of lice that are known to share their hosts with other feather louse ecomorphs tended to be more morphologically divergent (i.e. found in more disparate parts of morphospace; figure 2) than that of lice found as singletons (solo) on a host and occurring closer to morphological mean. Feather lice that share their host with other feather louse genera are also shown to exhibit significantly higher rates of morphological evolution than species that are known to be the only feather louse found on that host group.

Our results are supported by experimental evidence for louse–louse competition and microhabitat partitioning. For example, there is experimental evidence for competition for food resources between wing and body lice when host defences are removed [24]. Furthermore, Cicchino & Valim [37] compared louse oviposition between host species infected with single and multiple louse species. All three louse species laid their eggs on the head or neck of the bird when there were no other species present. However, when more than one species of louse was present on the same bird, each species laid its eggs in a more limited space, and the eggs of two different species were never found on the same feather in this case [37]. This behavioural flexibility of lice, in the presence of either host defences or competing louse species, could drive lice into different microhabitat niches [15]. Given that there are likely to be a limited number of microhabitats and mechanisms to escape from host preening on an individual bird, there is likely a relatively small number of ecomorphological types into which lice can diversify, leading to widespread convergence across clades.

In general, the evolution of permanent parasites is shaped by similar selective pressures to those in island model systems, particularly resource limitations. However, parasites face the additional pressure of dynamically evolving host defences against them. We found evidence that escaping from host defence and competition between lice likely act together to drive microhabitat partitioning and morphological evolution in feather lice. This partitioning ultimately results in convergence in morphology by the same specific morphological transitions in response to selection from the host's defence system. Overall, these results indicate that convergence in adaptation can also be a factor in driving divergence between related lineages. When the number of ecological outcomes is limited, as in the number of ways in which lice can escape host preening defences, this tends to favour convergence. Host-switching and open niches may also contribute to why feather lice show convergence, rather than evolutionary conservation, in body form.

We demonstrate the value of quantitative approach for identifying ecomorphological assignments in the future. Although the intensive effort and costly process to produce high-resolution 3D datasets limit the taxonomic and interspecific sampling of this study, the resulting volumes are data-rich, readily accessible, reusable and easy to incorporate into any future work. Future study should aim to increase inter- and intraspecific representation, incorporate additional louse traits (e.g. body shape), and incorporate host traits that are important for host defence to further understand the coevolutionary history between these two taxa.

## 4. Material and methods

### (a) Data collection and 3D scanning

We sampled 89 specimens representing 62 species of lice from all four ecomorphological groups (electronic supplementary material, datasets S1 and S2), designed to encompass the range of ecomorph variability and phylogenetic diversity in feather lice. Lice were identified to genus level using identification keys, and illustrations (e.g. *The chewing lice: world checklist and biological overview* [11]). Any species name attached to a specimen is based on previous sequencing and slide mounting determination from the same lot of specimens. In cases where only one species of a genus is known to parasitize a specific host, samples from that host species were given that tentative species determination. Initial ecomorph assignment followed previously published categorizations [9,14,15] and independent classification by other authorities [16]. Specimens had been previously collected from avian hosts by either using the postmortem ethyl acetate fumigation and ruffling method or dusting with pyrethrum powder for live birds [38] and stored in 95% ethanol at −80°C. We performed high-resolution X-ray computed tomography (CT) scanning at the University of Florida's Nanoscale Research Facility using Phoenix V|tome|x M (Waygate Technologies) and Xradia Versa 620 (Carl Zeiss optics) nanotomography systems. To retain the structural integrity of the mounting system while minimizing X-ray attenuation through excess or dense material, specimens were gently affixed to the surface of a length of low-density adhesive tape, which was then rolled into a tube and attached to a 3.5 mm diameter carbon fibre rod. This technique allowed up to six specimens to be mounted at once and scanned in sequence using the batch function of the Phoenix system and multiple recipes for the Versa. X-ray tube voltage was kept low (40–50 kV) to improve contrast, and geometric magnification (Phoenix) and a combination of geometric and optical magnification with a 4× objective (Xradia) was employed to minimize the voxel size and facilitate the recovery of microanatomy. Following scanning, the samples were gently removed from the adhesive tape by submerging them in alcohol. Radiographs were converted to tomograms using filtered back projection, with Datos|X R (Waygate Technologies) and Reconstructor Scout-and-Scan Control System (Zeiss) software.

CT scans were processed with VGStudioMax v. 3.5.5, using specific grey values of density to create 3D voxel regions of (1) the full body, (2) the head, (3) the chewing muscles of the specimens to obtain their volumetric (cubic) values (*n* = 79 specimens), and (4) lengths of leg segments. 3D shape files of the head were saved as mesh files in the community standard Polygon file format (.ply) for geometric morphometric analysis (*n* = 88 specimens). The files were then downsized in MeshLab v. 20.7 using the quadric-based edge collapse strategy. Finally, we collected surface measurements to obtain the width of the metanotum (figure 5*a*), and the lengths of the femur and tibia for each leg (*n* = 87 specimens); one low-quality image was removed owing to uncertainty in measurement precision. Tomograms of all datasets are freely available at Morphosource.org (project ID: 000610698; https://www.morphosource.org/projects/000610698).

### (b) Volumetric and measurement data

As all specimens were preserved in 95% ethanol, some desiccation of the soft tissue was apparent, and this impeded any comparative assessments of the shape of the soft abdomen. However, the thickened chitinous exoskeleton of the head, thorax and legs limited distortion in these areas and, while the cross-sectional area of chewing muscles was clearly impacted by the preservation, we observed a similar degree of myological contraction across all datasets. As the impact of ethanol storage appeared to be comparable across all samples, proportional volumetric data of chewing muscles were used to explore the differences between the ecomorph groups. To obtain the proportional values for each specimen, we divided the volume of the chewing muscles by the total volume of the head. Similarly, we used length values of both the femur and tibia individually and together and divided each by the width of the metanotum to obtain proportional length values. To determine if the values were normally distributed, we used a Shapiro–Wilk test of normality and found that neither chewing muscle volume (*p* < 0.05, *W* = 0.96) nor leg measurements (conducted for each leg individually, *p* < 0.05, *W*_leg1_ = 0.96, *W*_leg2_ = 0.91, *W*_leg3_ = 0.92) were normally distributed. Therefore, we selected the non-parametric Kruskal–Wallis (KW) rank sum test and Dunn's *post hoc* test for the following analyses. We looked for statistically significant differences between the ecomorph groups and sex using a KW test. To identify statistically significant differences, we used Dunn's test and a two-sided *p*-value with a Bonferroni correction using the R package ‘GmAMisc’ and function kwPlot (electronic supplementary material, figures S2–S4). Finally, the results of the volumetric dataset were compared with a phylogenetic ANOVA, using phylogenetic information (below) and adjusted *p*-value by means of a Bonferroni correction for multiple tests.

### (c) Phylogenetic information

We followed Johnson *et al*. [39] for genomic sequencing of lice. Specifically, for each louse species and 25 outgroup taxa (Anoplura, Trichodectera, Rhynchophthirina and Amblycera) (electronic supplementary material, dataset S2), we did single specimen-based genomic DNA extractions, prepared Illumina libraries, and sequenced them on an Illumina NovaSeq sequencer to achieve at least 30–60× coverage of the nuclear genome. We then pre-processed data using fastp v. 0.20.1 (phred quality ≥ 30, [40]) and deposited the raw reads in NCBI SRA (electronic supplementary material, dataset S2).

We used aTRAM v. 2.0 [41,42] to assemble a target set of 2395 single-copy orthologue protein-coding genes, using a panel of amino acid sequences from the human head louse *Pediculus humanus* as the reference. After assembly, we removed intron sequences using an Exonerate-based [43] stitching pipeline (atram_stitcher) that identifies exon sequences and stitches them together [44]. The whole assembly and phylogenomic pipeline, including processing steps, parameters and commands, followed previous studies [39,45].

We translated nucleotide sequences into amino acids and aligned them using MAFFT v. 7.471 [46]. After that, we back-translated sequences to nucleotides and used trimAL v. 1.4 (40% gap threshold) to trim individual gene alignments [47]. We discarded any gene that was represented by fewer than four taxa. We then concatenated gene alignments into a supermatrix and ran an IQ-TREE 2 v. 2.1.2 partitioned analysis that included model selection for each partition [48–51]. We estimated support using ultrafast bootstrapping in IQ-TREE [52,53]. We rooted the tree on Amblycera based on prior studies [10,45].

Lastly, we produced an ultrametric tree using the least-square dating (LSD2) method implemented in IQ-TREE [54]. For this analysis, we used the same calibration points as in Johnson *et al*. ([45] (root age 92 Ma) and set a minimum branch length constraint (*u* = 0.01) to avoid collapsing short but informative branches without introducing bias to the time estimates (see https://github.com/tothuhien/lsd2). Both the partitioned consensus tree and time-calibrated tree are available in the electronic supplementary material, dataset S3. For better visualization, we colourized the phylogenetic tree branches and tip names according to the ecomorph groups (figure 1). Additionally, we used the phytool function ‘bind.tip’ in R [55], to split the tree tips to have both female and male represented and corresponding to the morphological dataset for further analyses.

### (d) Geometric morphometric analyses of 3D landmark data

To analyse the head shape between the ecomorph groups, we used 3D shape files in the R package ‘geomorph’ [56], using our modified R script from Paluh *et al*. [57] (see electronic supplementary material or https://doi.org/10.5281/zenodo.8102696). Fourteen homologous landmarks were placed onto shape files in the same order to capture the shape variability of the head (electronic supplementary material, figure S5*a*,*b*). Then we turned these data into a 3D array for a generalized Procrustes analysis (GPA; electronic supplementary material, figure S5*c*) to obtain shape variables from landmark data. Each point was rotated and aligned, and Procrustes coordinates were produced using the 'geomorph' package (https://CRAN.R-project.org/package=geomorph) [56,58]. Additionally, we visualized the estimated mean shape from our data (electronic supplementary material, figure S5*d*).

We ran a principal component analysis (PCA), and the Procrustes-aligned specimens were plotted in three dimensions of tangent space for 88 specimens (PC1, PC2; figure 2). The first two principal components from the geometric morphometric analysis explain 63.99% variation in head shape (PC1 = 53.5%, PC2 = 10.49%). We used our phylogenetic tree with split tips to account for both males and females (*n* = 88) and ran the following analyses for 10 000 iterations. First, we ran a phylogenetic ANOVA to analyse the patterns of shape variation and covariation between ecomorphs in our set of aligned coordinates (running separately for PC1 and PC2). Next, we compared the net rates of morphological evolution of ecomorph groups and of solo versus co-occurring species using the simulation method for both, which uses the common evolutionary rate matrix for all species and with the multi-dimensional rate along the diagonal elements [59]. The function for permuting the Procrustes shape data among the tips of the phylogeny was used to identify phylogenetic signal using the *K*-statistic (*K* < 1 less phylogenetic signal than expected; *K* > 1 greater phylogenetic signal than expected under the Brownian motion model of evolution [60]). Additionally, a phylogenetic morphological PCA was conducted (figure 3), with estimated ancestral states and phylogenetic branches projected into ordination plots [61]. We used two approaches: (1) a phylogenetic PCA (phy-PCA; figure 3*a*,*c*), which reveals trends that are the most independent of phylogenetic variation; and (2) a phylogenetically aligned PCA (PACA; figure 3*b*,*d*), which reveals the variation that is most associated with the phylogenetic signal. Additionally, we used both variants for centring and projections via ordinary least squares (OLS; figure 3*a,b*) and GLS (figure 3*c,d*) [61].

## Data Availability

The datasets and code used during the current study are available at Zenodo (https://doi.org/10.5281/zenodo.8102696) [62]. We used museum-collected specimens and their IDs are included in electronic supplementary material, tables S1 and S2. Tomograms of all datasets are freely available at Morphosource.org (project ID: 000610698; https://www.morphosource.org/projects/000610698). Supplementary material is available online [63].
